# Inclusive Medical Spanish Education to Address Bias in Clinical Communication: Tutorial on Patient-Informed Module Development

**DOI:** 10.2196/98398

**Published:** 2026-08-21

**Authors:** Alexandra Lopez Vera

**Affiliations:** 1 California University of Science and Medicine, 1501 Violet St, Colton, CA, 92324, United States, 1 5628524232

**Keywords:** medical Spanish, inclusive communication, LGBTQ+ health, bias, clinical communication, language concordance, standardized patients, simulation training, digital education, curriculum development

## Abstract

Spanish-speaking patients who are lesbian, gay, bisexual, transgender, queer, and other minority sexual orientations and gender identities (LGBTQ+) may encounter overlapping barriers in clinical care related to language discordance, misgendering, heteronormative questioning, and culturally unresponsive communication. These barriers can undermine trust, limit disclosure, and negatively affect care experiences and continuity of care. At the same time, medical Spanish curricula in the United States remain highly variable in content, structure, and assessment, and inclusive, bias-aware communication has not been consistently integrated into language training for health professions learners. Our prior qualitative work with Latinx, Hispanic, and Spanish-origin LGBTQ+ adults identified communication priorities directly relevant to educational design, including respectful forms of address, open-ended relationship language, broader recognition of family structures, and reduced reliance on binary or heteronormative assumptions. Our prior curricular work has also shown that structured medical Spanish instruction using self-study, faculty-led teaching, peer support, standardized patient encounters, and performance-based assessment can be implemented in undergraduate medical education. This tutorial presents a patient-informed framework for developing an inclusive medical Spanish module to address bias in clinical communication. Rather than reporting a new intervention study, it synthesizes prior qualitative findings and prior curricular experience to provide practical guidance for educators. The framework describes how to translate patient-reported communication priorities into curricular design principles, learning objectives, technology-enhanced preparatory activities, standardized patient and live-practice components, feedback and assessment strategies, and implementation planning. It argues that inclusive communication should not be treated as optional cultural content or as a narrow language add-on but as a core component of competent clinical performance in Spanish-language care. This tutorial is intended for medical educators, language educators, course directors, and simulation teams seeking to strengthen language-concordant and bias-aware communication training in medical Spanish. By linking patient-informed findings to concrete educational design choices, the framework offers a practical model for integrating inclusive communication throughout instruction, simulation, and assessment. A patient-informed approach of this kind may help health professions programs move beyond general statements about inclusion and toward more deliberate preparation of learners to communicate respectfully and effectively with Spanish-speaking LGBTQ+ patients.

## Introduction

Bias in health care interactions can be reinforced through gendered assumptions and norms that shape how patients are perceived and treated [1]. For lesbian, gay, bisexual, transgender, queer, and other minority sexual orientations and gender identities (LGBTQ+) patients, noninclusive communication, misgendering, and other forms of exclusion may negatively affect disclosure, trust, and overall care experiences [2-6]. These challenges may become even more complex when patients must also navigate sensitive clinical conversations across language barriers [7].

This issue is especially relevant in the context of Spanish-speaking patient care. Medical Spanish education has expanded across US medical schools in response to the growing need for language-concordant care, yet programs remain highly variable in content, structure, and assessment [8-11]. Prior scholarship has shown that medical Spanish curricula can improve learner confidence, vocabulary, and communication skills through approaches such as didactic instruction, role-play, standardized patient encounters, immersion, and systems-based teaching [8-13]. However, the integration of inclusive, bias-aware communication into these curricula remains limited, despite increasing recognition that many trainees feel underprepared to care for transgender and gender-diverse populations and that this area remains insufficiently addressed in medical education [14-17]. National practice-level data reinforce this gap: in a 2025 survey of 1245 US primary care practices, most practices collected patient data on gender identity (77%) and sexual orientation (76%), yet only 34% provided LGBTQ+-focused training for clinicians, suggesting that data collection has outpaced workforce preparation for affirming communication [18].

For Spanish-speaking LGBTQ+ patients, communication barriers are not solely linguistic. They are also shaped by the intersection of gender identity, cultural context, and clinical assumptions. In our prior qualitative work, Latinx, Hispanic, and Spanish-origin LGBTQ+ adults described several recurring concerns in health care interactions, including misgendering, binary assumptions in routine questioning, noninclusive language related to sexual practices and relationships, and a lack of culturally responsive, language-concordant care [2]. The qualitative study informing this tutorial included 28 Latinx, Hispanic, and Spanish-origin LGBTQ+ adults between the ages of 19 and 43 years who participated in brief, semistructured interviews about gender-affirming language in Spanish-language clinical communication [2]. Participants were recruited through community organizations, health care providers, and word-of-mouth within Latinx, Hispanic, and Spanish-origin communities, and interviews were conducted in person, by phone, or by videoconferencing according to participant preference [2]. The study examined participants’ experiences with pronoun use, binary and heteronormative language, relationship and family terminology, sexual health communication, and culturally responsive language-concordant care [2]. Participants also described clear preferences for affirming pronoun use, open-ended relationship language, broader recognition of family structures, and communication practices that reduce the burden of repeatedly explaining identity within the medical encounter [2].

In this tutorial, open-ended relationship language refers to Spanish phrasing that allows patients to describe their relationships, partners, and support systems in their own terms rather than requiring them to fit into presumed categories such as *esposo*/*esposa*, *novio*/*novia*, or *madre/padre*. In clinical communication, this includes using broader terms such as *pareja*, *persona de apoyo*, or *familia/red de apoyo* when the patient’s relationship structure or preferred terminology has not yet been established. This approach is intended to reduce heteronormative and family-structure assumptions while preserving clinical clarity. These findings suggest that medical Spanish training should move beyond vocabulary acquisition alone and explicitly address how language choices can either reinforce or reduce exclusion in clinical care.

At the same time, our prior curricular work has demonstrated the feasibility of incorporating this content into structured medical Spanish instruction. Our previously published endocrine system module combined self-study, faculty-led sessions, standardized patient practice, peer tutor support, and performance-based assessment to help first-year medical students strengthen Spanish-language clinical communication while incorporating inclusive care principles [12]. That experience showed that blended, systems-based teaching models can provide a useful foundation for more intentional educational design in this area [12].

Despite these advances, educators still lack practical guidance for translating patient-informed findings on inclusive and bias-aware communication into a teachable and adaptable medical Spanish module. This gap is particularly relevant to current discussions in health professions education regarding bias, intersectionality, and the need for more intentional training approaches for inclusive care [10,14,16]. The purpose of this paper is therefore to present a patient-informed framework for developing a medical Spanish educational module that addresses bias in clinical communication with Spanish-speaking LGBTQ+ patients. Rather than reporting a new intervention study, this paper synthesizes prior qualitative findings and prior curricular experience to offer an educator-facing model for module design, implementation, and adaptation across training settings.

## Visual Framework Summary

**Figure 1.**
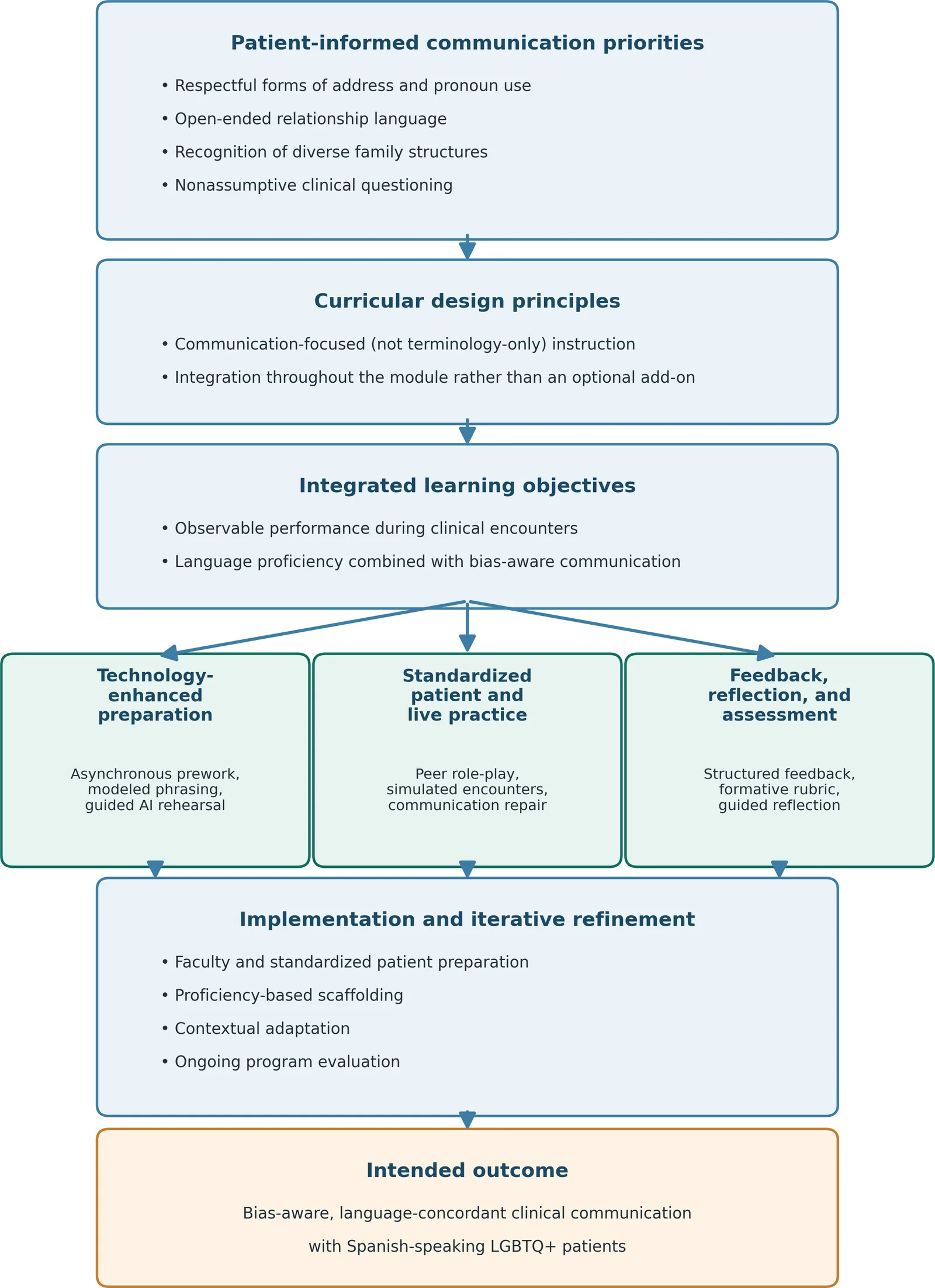
Visual summary of the patient-informed framework for developing an inclusive medical Spanish module. LGBTQ+: lesbian, gay, bisexual, transgender/transsexual, queer, and other minority sexual orientations and gender identities.

The framework presented in this tutorial can be summarized as a sequence of five linked components: (1) patient-informed communication priorities identified in prior qualitative work; (2) curricular design principles that treat inclusive communication as a clinical skill rather than an optional add-on; (3) integrated learning objectives that combine linguistic performance with bias-aware communication; (4) instructional delivery through technology-enhanced preparation, standardized patient and live practice, and structured feedback, reflection, and assessment; and (5) implementation planning with iterative refinement. Figure 1 presents this framework as a logic model linking patient-informed evidence to instructional design and to the intended outcome of bias-aware, language-concordant clinical communication.

## From Patient-Informed Findings to Curricular Design Principles

An inclusive medical Spanish module should begin with the recognition that communication failures in this area are not limited to vocabulary gaps. They are also shaped by assumptions embedded in routine clinical language, including assumptions about gender identity, pronoun use, sexual practices, relationships, and family structure [2-6]. For Spanish-speaking LGBTQ+ patients, these challenges may be amplified when language discordance intersects with culturally unresponsive care, making it insufficient to teach terminology or memorized question patterns alone [2].

Patient-informed findings should therefore serve as a central foundation for curricular design rather than as supplemental cultural content. Our prior qualitative work identified several recurring priorities that can be translated directly into educational goals [2]. First, learners must be prepared to avoid defaulting to binary language and to ask respectfully how patients would like to be addressed. Because Spanish is a highly gendered language, inclusive medical Spanish instruction must address more than pronoun use alone. In this tutorial, “defaulting to binary language” refers to the automatic use of gendered forms of address, relationship terms, family terms, grammatical agreement, and clinical categories that presume a patient is male or female, heterosexual, cisgender, or situated within a conventional family structure. Examples include relying on *señor*/*señora* before confirming how a patient wishes to be addressed, using *esposo*/*esposa* or *novio*/*novia* when pareja would allow broader self-description, or framing reproductive and anatomical questions in ways that equate gender identity with specific organs or reproductive capacity. This issue is especially complex in Spanish because gender marking is embedded across nouns, adjectives, articles, and many routine clinical phrases. Therefore, inclusive Spanish-language clinical communication should not be reduced to substituting one term for another. Instead, educators should teach learners to ask respectfully about preferred forms of address, use neutral or open-ended terms when patient preferences are unknown, apply anatomy-specific language when clinically relevant, and adapt phrasing based on the patient’s own terminology.

Second, they must learn to conduct relationship, sexual, and social history taking without presuming heterosexuality, monogamy, or conventional reproductive expectations [2-4]. Third, they must be able to use language that acknowledges broader family structures and diverse lived experiences while maintaining cultural and linguistic responsiveness [2]. These priorities are central to whether a clinical encounter feels respectful, accurate, and safe.

Educators should also anticipate regional and generational variation in Spanish itself. Gender-neutral innovations such as the pronoun *elle* and -e or -x word endings (eg, *Latine*, *Latinx*) are still evolving, and they are not universally adopted, understood, or welcomed across Spanish-speaking regions, dialects, and generations; some patients experience these forms as affirming, whereas others find them unfamiliar or externally imposed. Instructional guidance should therefore be explicit about how to navigate this variation in a clinical setting. Learners should be taught to follow the patient’s own terminology rather than to impose any particular form, to ask respectfully when uncertain, and to use rephrasing strategies that avoid gender marking altogether when preferences are unknown (eg, selecting verbs and sentence structures that do not require gendered agreement). Widely understood terms such as *pareja* or *persona de apoyo* provide a practical starting point across regions. At the program level, facilitator orientation should include a brief discussion of the regional and generational variation most relevant to the local patient population, and standardized patients, community advisors, and local language experts can help adapt phrase banks to the linguistic profiles of the communities each program serves [2,12].

Translating these findings into curricular design requires a shift from terminology-focused instruction to communication-focused instruction. Medical Spanish education has demonstrated value in improving learner confidence, vocabulary, and clinical communication through approaches such as didactic teaching, role-play, systems-based instruction, and standardized patient encounters [8-12]. At the same time, the literature shows that training related to transgender and gender-diverse care remains limited across medical education, and many learners continue to feel underprepared in this area [14-17]. In this context, an inclusive medical Spanish module should not be treated as a narrow language add-on. Rather, it should be framed as a structured response to an educational gap at the intersection of language, identity, and clinical bias.

In practical terms, this means that curricular design should be organized around communication tasks that commonly become sites of exclusion in clinical encounters. These include opening the visit, asking about names and pronouns, taking a sexual history, discussing partners, exploring family context, and eliciting social history. Each of these moments should be treated as a core teaching opportunity rather than as incidental cultural content. Cases and scenarios should require learners to apply inclusive language in realistic clinical interactions rather than limiting this content to isolated vocabulary lists or brief awareness discussions [2,12].

This approach also fits well within existing medical Spanish educational models. Learners benefit from structured sequencing, repeated exposure, and multiple opportunities to practice clinical communication through self-study, faculty-led teaching, peer-supported learning, and standardized patient encounters [8-12]. Building on that foundation, an inclusive module should be intentionally sequenced so that learners first develop the language needed for respectful and nonassumptive communication and then apply that language in progressively more complex clinical contexts. In this way, patient-informed curricular design becomes the bridge between language preparation and bias-aware clinical performance.

A brief clinical vignette illustrates how these design principles converge within a single, clinically ordinary encounter in which identity-related communication is integrated into a standard presenting complaint rather than being treated as the subject of the visit (Textbox 1).

Textbox 1. **Illustrative clinical vignette: integrating inclusive communication into a routine asthma follow-up visit.**

A second-year medical student conducts a Spanish-language follow-up visit for asthma. The student opens with “Buenos días, soy estudiante de medicina. ¿Cómo prefiere que le llame?” and learns that the patient goes by Alex. The visit proceeds as an ordinary follow-up: inhaler technique, symptom frequency, and nighttime awakenings. When reviewing triggers in the home environment, the student asks “¿Quién vive con usted?” rather than assuming “¿Está casada?,” and the patient describes a household that includes their partner and a cousin who smokes on the balcony—clinically relevant information for trigger reduction that a narrower, assumption-laden question might have missed. While updating the medication list, the student learns that the patient recently began gender-affirming hormone therapy at another clinic and acknowledges it briefly and respectfully—“Gracias por decirme; lo anoto para revisar sus medicamentos”—without making identity the focus of the visit. The encounter closes with an updated asthma action plan. Identity is never the chief complaint; it surfaces naturally, is handled respectfully, and improves the clinical quality of the visit. Full standardized patient cases built on this structure, including expected communication behaviors and common learner errors, are provided in Multimedia Appendices 1 and 2.

## Learning Objectives for an Inclusive Medical Spanish Module

Learning objectives for an inclusive medical Spanish module should reflect both linguistic performance and clinical communication. If the objectives focus only on vocabulary or grammatical accuracy, they risk overlooking the very forms of exclusion the curriculum is intended to address. Communication barriers related to misgendering, heteronormative assumptions, and limited recognition of diverse family and relationship structures are well documented [2], suggesting that these areas should be treated as core instructional targets rather than as secondary cultural considerations. These objectives should also remain consistent with the broader medical Spanish literature, which shows that communication skills are most effectively developed when language instruction is embedded in clinically meaningful contexts [8-12].

One principle is especially important: objectives should be written around what learners must be able to do during an encounter, not simply what they must know. In this context, knowledge of inclusive terminology is important, but it is not sufficient. Learners must be able to introduce themselves respectfully, ask how patients would like to be addressed, obtain histories without relying on heterosexual or binary assumptions, and navigate identity-sensitive topics in Spanish with clarity and professionalism [2,12]. Framing objectives in this way keeps the curriculum grounded in observable performance rather than abstract awareness alone.

A second principle is that objectives should integrate language and bias-responsive care rather than separating them into different parts of the module. In many educational settings, language instruction and equity-oriented content are delivered on parallel tracks. For this topic, such separation is counterproductive. The aim is not simply to teach learners about inclusion and then separately teach them Spanish. It is to help them use Spanish in ways that reduce exclusion and improve the quality of clinical communication. For that reason, objectives should consistently reflect both the communicative function and the relational intent [10,12].

The following objectives may serve as a practical starting point for module design:

1. Use respectful Spanish introductions and ask patients how they would like to be addressed, including a discussion of name and pronoun preferences when clinically appropriate [2-4].
2. Conduct relationship, sexual, and social history taking in Spanish without assuming heterosexuality, monogamy, a binary gender identity, or conventional reproductive goals [2-4,12].
3. Use open-ended and inclusive Spanish language to discuss partners, family structures, and lived experiences relevant to patient care [2,4].
4. Recognize how routine clinical phrasing can communicate exclusion and revise questions in ways that are more affirming, accurate, and patient centered [2].
5. Apply inclusive communication principles in simulated clinical encounters with Spanish-speaking patients in a manner that is linguistically appropriate and culturally responsive [2,12,13].
6. Demonstrate increasing comfort with identity-sensitive communication in Spanish through repeated practice, feedback, and guided reflection [8-12].

Programs may adapt these objectives depending on learner proficiency, available instructional time, and the clinical focus of the module. For novice learners, emphasis may be placed on introductions, names and pronouns, basic nonassumptive history questions, and simple communication repair when a linguistic or interpersonal misstep occurs. For more advanced learners, the module can extend to more complex discussions of sexual health, family context, counseling, and more nuanced repair strategies. Communication repair should therefore be introduced across proficiency levels rather than reserved for advanced learners, with expectations scaffolded according to linguistic complexity. In either case, the objectives should remain closely tied to the communication priorities identified by patients and to the practical demands of real clinical encounters [2,12].

Well-designed objectives also support alignment across the rest of the curriculum. Once the objectives are clearly defined, they can guide the selection of digital prework, live teaching content, standardized patient scenarios, peer tutor activities, and performance assessments. In this way, the objectives become more than a formal requirement; they become the organizing framework for a module that is both educationally coherent and responsive to documented gaps in clinical communication training [12].

## Technology-Enhanced and Preparatory Components of an Inclusive Medical Spanish Module

An inclusive medical Spanish module is well suited to a technology-enhanced format because learners benefit from repeated exposure to terminology, phrasing, and clinical scenarios before they are asked to perform in real time. In our prior curricular work, structured self-study paired with faculty-led teaching and simulation-based instruction supported vocabulary development, learner confidence, and clinical communication [11,12]. In this context, preparatory activities should do more than preview content. They should help learners arrive with sufficient familiarity to focus during live sessions on respectful language use, nonassumptive questioning, and communication repair.

Preparatory components should, therefore, be designed around high-yield communicative tasks rather than broad content review alone. Before a live session, learners may complete short asynchronous activities introducing inclusive greetings, questions about names and forms of address, open-ended relationship language, and sexual history phrasing that does not presume binary identity or a heterosexual partnership [12]. These materials can also introduce broader family language and examples of how small wording changes may shape the tone and inclusivity of a clinical encounter [2]. At this stage, the goal is not mastery. It is to provide enough baseline familiarity so that live instruction can focus on application rather than first exposure.

Technology-enhanced preparation can take multiple forms depending on institutional resources. Practical options include narrated slide presentations, short instructional videos, online glossaries, digital case prompts, learning management system modules, and recorded demonstrations of inclusive and noninclusive communication. These formats are consistent with the broader direction of medical Spanish education, which has incorporated multiple instructional modalities to support communication training [11-13]. In a module focused on inclusive and bias-aware communication, digital content should be selected not only for convenience but also for its ability to model language in context. Learners should be able to hear and see how questions are framed, how clarifying language can be used respectfully, and how patient-centered phrasing differs from more exclusionary defaults. Table 1 provides concrete examples of technology-enhanced preparatory activities, the communication focus of each, and practical implementation notes.

**Table 1.** Examples of technology-enhanced preparatory activities for an inclusive medical Spanish module.

Preparatory activity	Example	Communication focus
Narrated microlessons or short instructional videos	About 5- to 10-minute recordings that model respectful openings and preference-checking questions (eg, “¿Cómo prefiere que le llame?")	Forms of address; avoiding automatic señor/señora defaults
Recorded contrast demonstrations	Paired clips of the same intake performed with assumptive versus nonassumptive phrasing, followed by brief comparison questions	Recognizing how routine phrasing communicates inclusion or exclusion
Digital phrase banks and glossaries	Searchable phrase lists organized by communicative function (forms of address, relationships, family and support systems, sexual history, communication repair) rather than alphabetically	Open-ended relationship, family, and support-system language
Learning management system case prompts	Short written vignettes that ask learners to draft or select the next question in Spanish, with automated or faculty feedback	Nonassumptive history taking with clinical specificity
Faculty-guided AI conversational rehearsal	Structured prompts for rehearsing Spanish-language history questions with a large language model, with explicit safeguards (no real patient information, faculty-reviewed prompt sets, and reminders that AI feedback may be inaccurate or culturally limited)	Low-stakes rehearsal of phrasing, tone, and communication repair

Emerging AI tools, including large language models and conversational agents, may also support preparatory practice when used with clear instructional boundaries. For example, learners may use faculty-guided AI prompts to rehearse Spanish-language clinical questions, receive formative feedback on tone and phrasing, compare assumptive and nonassumptive wording, or practice communication repair after a linguistic or interpersonal misstep. However, AI-based practice should not replace faculty instruction, standardized patient encounters, or expert feedback. Programs should establish safeguards related to accuracy, privacy, bias, and oversight, including prohibiting the entry of real patient information, reviewing AI-generated language for clinical and linguistic appropriateness, and making clear to learners that AI feedback may be incomplete, culturally limited, or inaccurate.

Preparatory work should also be sequenced intentionally. One practical approach is to begin with foundational terminology and short phrase sets, then move to brief clinical exchanges, and finally introduce more complex scenario-based prompts. For example, learners may first review Spanish expressions for introductions and patient comfort, then practice asking about partners or family in more open-ended ways, and only afterward work through a short clinical vignette that requires them to integrate several elements into a single interaction [2,12]. This progression is particularly useful for learners managing the combined demands of language production and clinical reasoning.

Importantly, preparatory materials should not isolate inclusive communication as a purely cultural add-on. If this content appears only in a side module or optional reading, learners may interpret it as secondary rather than clinically essential. Instead, inclusive and bias-aware communication should be embedded directly into the same materials learners use to prepare for history taking, counseling, and standardized patient encounters [2,12,13]. This helps frame inclusive language not as a special topic separate from clinical skill but as part of competent clinical communication itself.

When possible, preparatory activities should also create continuity with later in-person or synchronous instruction. The same terminology, scenarios, and communicative goals introduced asynchronously should reappear during faculty-led teaching, peer-supported practice, and simulated encounters. This alignment reinforces learning and helps ensure that digital preparation is not perceived as disconnected homework but as the first stage of a coherent instructional sequence [11-13]. In a patient-informed module, technology-enhanced preparation is most effective when it serves as a bridge between foundational language review and the more demanding interpersonal work of live clinical communication.

## Standardized Patient and Live Practice Components of the Module

Live practice is essential in an inclusive medical Spanish module because many of the communication challenges identified in prior work arise during real-time interaction rather than in isolated vocabulary recall [12,13]. Misgendering, heteronormative questioning, and noninclusive assumptions about family or sexual practices do not usually occur because learners lack a single term. Rather, they emerge when learners must make rapid linguistic and interpersonal choices during a clinical encounter [2-4]. For this reason, the live portion of the module should be designed to help learners apply inclusive language within realistic clinical tasks rather than simply repeat prepared phrases.

Standardized patient encounters are particularly useful for this purpose. Our prior curricular experience demonstrated the value of structured practice with standardized patients and peer-supported rehearsal as part of systems-based instruction [11-13]. In an inclusive module, these encounters should be designed so that bias-aware communication is not peripheral to the case but integral to it. Learners should need to ask how a patient would like to be addressed, avoid assumptions about partner gender or relationship structure, and navigate identity-sensitive history taking in Spanish as part of the encounter itself [2,12]. This helps ensure that inclusive communication is taught as a component of competent clinical practice rather than as an optional addition after the main interaction.

Case design is therefore critical. A well-designed standardized patient scenario should create authentic opportunities for learners to practice inclusive language in context. This may occur during the opening of the encounter, the social history, the sexual history, or discussions involving family, support systems, or prior experiences with health care. Cases should be written carefully so that learners must gather clinically relevant information without relying on binary or heteronormative defaults [2-4]. At the same time, the case should remain clinically coherent and should not reduce LGBTQ+ identity to the sole focus of the encounter. The goal is not to create “identity cases” but to normalize inclusive communication within ordinary patient care.

Small-group live practice can further strengthen this part of the module. Learners benefit from opportunities to rehearse language with faculty, peer tutors, or both before and after standardized patient encounters [12,13]. These sessions allow learners to test phrasing, receive immediate correction, and build confidence before higher-stakes performance. In an inclusive medical Spanish module, small-group practice should focus not only on whether a sentence is grammatically correct but also on whether it is respectful, open-ended, and clinically appropriate. This distinction matters because a grammatically correct question can still communicate exclusion if it embeds the wrong assumptions.

Feedback during live practice should be structured and specific. Learners should receive guidance on forms of address, question framing, avoidance of assumptions, communication repair, and overall patient-centeredness [2,12]. Feedback is especially important when learners make errors that may not be obvious to them, such as shifting into binary phrasing after an inclusive introduction or asking follow-up questions that quietly reintroduce heterosexual assumptions. Correcting these patterns in real time can help learners understand that inclusive communication depends not only on isolated terminology but also on consistency across the encounter.

Whenever possible, the standardized patient and live practice components should be sequenced from lower-stakes to higher-stakes interactions. Learners may first practice short exchanges in pairs or small groups, then move into longer simulated encounters that require greater fluency, flexibility, and clinical reasoning [11-13]. This progression supports skill development while helping learners manage the communicative demands of the encounter more effectively. In a patient-informed module, live practice is most effective when it gives learners repeated opportunities to use inclusive Spanish in realistic settings, make mistakes, receive correction, and try again under guided conditions.

To make the tutorial more directly usable for educators, supplementary teaching materials that translate the framework into sample instructional tools are provided. Multimedia Appendix 1 includes a sample patient case focused on forms of address, pronoun-related communication, and inclusive intake language. Multimedia Appendix 2 includes a sample patient case focused on relationship language, sexual history-taking, and avoidance of heteronormative assumptions. Multimedia Appendix 3 includes a facilitator guide and sample assessment rubric that can be adapted for peer role-play, standardized patient encounters, or formative assessment. These materials are intended as adaptable examples rather than fixed scripts, allowing programs to modify the level of linguistic complexity, clinical focus, and assessment expectations according to learner proficiency, curricular time, and local implementation needs.

## Feedback, Reflection, and Assessment Strategies

Assessment in an inclusive medical Spanish module should extend beyond whether learners can recall vocabulary or complete a checklist of phrases. If the goal is to prepare learners for more respectful and bias-aware clinical communication, assessment must capture how language is used in interaction, how questions are framed, and whether learners can sustain inclusive communication across the encounter [2-4]. This is especially important because some of the most consequential communication failures in this area do not stem from the absence of Spanish proficiency but from the persistence of binary, heteronormative, or culturally unresponsive assumptions within otherwise fluent interactions [2].

For that reason, feedback should be built into the module as an ongoing instructional component rather than reserved for a final evaluation. Structured practice; performance-based assessment; and feedback from faculty, peer tutors, and standardized patients have all demonstrated value in medical Spanish education, including in our own curricular experience [11-13]. In an inclusive medical Spanish module, feedback should address both linguistic and interpersonal dimensions of performance. Learners may need correction not only on vocabulary choice or grammar but also on how they introduce themselves, whether they ask about names and forms of address appropriately, whether they default to binary or heteronormative assumptions, and whether they adjust their phrasing respectfully when clarification is needed [2,12].

Reflection can further strengthen this process. Because bias in communication is often reproduced through habitual phrasing, learners may not immediately recognize where exclusion has occurred in their own language use. Short reflective activities after simulated encounters can help learners identify moments in which their wording narrowed the encounter, introduced assumptions, or failed to align with patient preferences [2-4]. Reflection should not be treated as a substitute for skill practice, but it can help learners connect specific language choices with broader relational and clinical consequences. In this way, reflection supports the module’s purpose of moving learners from awareness to more intentional communication.

Because learners at all proficiency levels may make errors in gendered or relationship language, feedback should explicitly include how learners recognize, acknowledge, and repair missteps in Spanish. At earlier levels, this may involve brief acknowledgments and clarification requests; at more advanced levels, learners can be expected to repair the interaction while maintaining the flow of clinical history taking and patient-centered communication.

Assessment strategies should be aligned directly with the module’s learning objectives. If the objectives include asking respectfully about names and pronouns, conducting nonassumptive histories, and using more inclusive language around relationships and family, then those elements should appear explicitly in assessment tools [2,12]. Programs may choose to assess confidence, knowledge, and performance at different points in the module. For example, confidence surveys can help identify perceived growth in comfort with inclusive Spanish communication, whereas written or digital exercises can assess comprehension of key terminology and phrasing [12]. Performance-based assessments, however, are especially important because they allow educators to evaluate whether learners can apply these skills during interactions rather than only recognize them in theory.

Standardized patient encounters, observed role-play, and oral assessments are particularly well suited to this purpose [8-13]. These formats allow educators to evaluate not only what learners ask, but how they ask it and whether their communication remains respectful and open-ended throughout the encounter. In an inclusive medical Spanish module, assessment rubrics should therefore include criteria related to inclusive introductions, avoidance of unsupported assumptions, appropriateness of follow-up questioning, consistency in address language, and overall patient-centeredness [2,12]. Table 2 presents a simplified formative assessment rubric organized around these domains; a complete facilitator guide and expanded rubric are provided in Multimedia Appendix 3. Even when formal grading is limited, these domains can be incorporated into formative rubrics to guide feedback and make expectations explicit.

**Table 2.** Simplified formative assessment rubric for inclusive Spanish-language clinical communication.

Domain	Developing	Competent	Strong
Respectful opening and form of address	Relies on visual assumptions or inconsistent forms of address	Asks or confirms how the patient wants to be addressed and generally follows the preference	Consistently uses patient-centered forms of address from the start of the encounter
Nonassumptive relationship and family language	Uses gendered or heteronormative terms without confirming relevance	Uses broader terms (eg, pareja, red de apoyo) when preferences are unknown	Uses open-ended language naturally and follows the patient’s own terminology throughout
Sexual history and clinical specificity	Avoids sensitive questions or embeds assumptions about partners or practices	Asks relevant questions with mostly inclusive, clinically clear phrasing	Balances inclusive language with precise behavior-specific and anatomy-specific questions
Communication repair	Does not recognize or repair a misstep or becomes defensive	Acknowledges correction and makes a basic repair	Repairs briefly and respectfully, then continues without centering the mistake
Spanish language effectiveness	Meaning is frequently unclear or dependent on English	Spanish is understandable and adequate for the task	Spanish is clear, organized, and appropriate to the clinical task and learner level
Patient-centeredness	Interaction feels checklist-driven or assumptive	Maintains a respectful tone; patient can provide relevant information	Creates an interaction in which the patient can self-describe and participate comfortably

Importantly, assessment in this context should aim to support growth rather than punish hesitation. Many learners will be practicing language that feels unfamiliar while also trying to avoid deeply normalized clinical defaults. A well-designed module should therefore create opportunities for low-stakes feedback before high-stakes performance. This allows learners to revise phrasing, improve consistency, and build confidence over time [8-13]. In a patient-informed and bias-responsive curriculum, the purpose of assessment is not only to document performance, but to help learners develop more respectful and clinically effective communication practices through repeated practice, targeted correction, and reflection.

## Implementation Considerations and Faculty Preparation

One major consideration is faculty preparation. Instructors, peer tutors, and standardized patients should be oriented not only to the linguistic content of the module but also to its communication goals. Medical Spanish instruction often relies on multiple teaching formats and facilitators, which can strengthen learning but also create variation in how content is presented [8-12]. In an inclusive module, facilitator preparation is particularly important because learners are likely to notice inconsistencies quickly. If inclusive language is emphasized in one part of the module but absent or handled hesitantly in another, the curriculum may inadvertently signal that bias-aware communication is optional or secondary. Faculty development should therefore include guidance on core terminology, question framing, case goals, and approaches to correcting learner language in ways that are clear, respectful, and educationally constructive [2,12].

To help programs budget administrative resources, approximate time requirements can be estimated as follows. Faculty and peer-tutor orientation can usually be accomplished in a 1- to 2-hour workshop covering module goals, core phrase categories, anticipated learner errors, and rubric use; a half-day session is preferable when facilitators are new to LGBTQ+ health content or when multiple new cases will be piloted. Standardized patient training typically requires an additional 2 to 3 hours per case, including case familiarization, rehearsal of responses to assumptive versus nonassumptive questioning, and calibration of feedback. Initial development or local adaptation of preparatory materials and cases commonly requires 10 to 20 hours of faculty time, which can be substantially reduced by adapting existing materials such as those provided in Multimedia Appendices 1-3.

Learner discomfort with identity-sensitive communication deserves particular attention because topics involving gender identity and sexual orientation can encounter personal, cultural, or political resistance from some learners. Several concrete strategies can help facilitators mitigate this discomfort. First, framing matters: the module should be introduced explicitly in terms of patient safety, rapport, diagnostic accuracy, and clinical evidence—learners are being trained to obtain complete and accurate histories and to avoid communication failures that reduce disclosure—rather than as ideological content. Second, facilitators should normalize discomfort openly, acknowledging that practicing unfamiliar phrasing in a second language feels awkward for nearly all learners and should model making and repairing errors themselves. Third, when defensive reactions arise during discussion, facilitators can redirect them to the clinical task (eg, “How would you ask this so the patient can give you accurate information?") rather than debating personal beliefs, keeping attention on observable communication behaviors. Fourth, low-stakes sequencing—pair practice before group performance, and formative feedback before any graded assessment—reduces the social risk of experimentation. Finally, ground rules established at the outset, including respectful language, confidentiality of practice-room errors, and feedback directed at behavior rather than character, create the predictability that supports engagement. These strategies allow programs to preserve the module’s goals while meeting learners where they are [2,12,13].

Because institutions differ in faculty expertise, learner proficiency, curricular time, simulation resources, and community partnerships, implementation should be planned with anticipated barriers in mind. Table 3 summarizes common challenges and practical strategies for adapting the module across educational settings. These strategies are intended to help programs preserve the core communication goals of the module while adjusting the instructional format to local resources and constraints.

**Table 3.** Anticipated implementation challenges and practical strategies.

Implementation challenge	Practical strategy
Limited faculty expertise in both medical Spanish and LGBTQ+^a^ health	Use a coteaching model when possible, pairing medical Spanish educators with faculty or staff who have expertise in LGBTQ+ health. If co-teaching is not feasible, provide facilitator scripts, phrase banks, and a brief orientation guide before implementation [2,8-12].
Wide variation in learner Spanish proficiency	Use scaffolded materials that address the same communication goal at different proficiency levels. Novice learners can focus on respectful introductions and basic open-ended phrasing, whereas advanced learners can practice more complex sexual history, counseling, and communication repair [8-13].
Limited curricular time	Move terminology, short explanations, and preparatory examples to asynchronous prework. Reserve synchronous time for role-play, standardized patient practice, feedback, and reflection [11-13].
Lack of trained standardized patients	Begin with peer role-play, scripted cases, or facilitator-led practice. Standardized patients can be added later once case goals, feedback expectations, and assessment criteria are established [12,13].
Learner discomfort or fear of making mistakes	Normalize error and repair as part of language learning. Introduce brief repair phrases early and create low-stakes practice opportunities before formal assessment [2,12,13].
Inconsistent facilitator feedback	Use a shared facilitator guide and rubric so that instructors, peer tutors, and standardized patients evaluate the same domains, including forms of address, nonassumptive questioning, relationship language, and patient-centeredness [2,12,13].
Institutional discomfort with inclusive language content	Frame the module as patient-centered clinical communication, accuracy, trust-building, and reduction of assumptions in care, rather than as optional cultural content [2-4,10,14-17].
Limited community partnerships	Use patient-informed literature, published cases, and faculty-reviewed materials initially, while developing partnerships with community advisors or LGBTQ+ health organizations over time [2,12,13].
Risk of treating LGBTQ+ identity as the entire case	Design cases in which identity is relevant to communication but not the only clinical issue. This helps normalize inclusive communication as part of routine care rather than presenting it as a separate diversity exercise [2-4,12].
Sustainability over time	Collect learner feedback, facilitator feedback, and performance data after each implementation cycle. Revise the module iteratively instead of treating it as a one-time workshop [8-13].

a LGBTQ+: lesbian, gay, bisexual, transgender, queer, and other minority sexual orientations and gender identities.

A second consideration is learner proficiency. Medical Spanish courses often include students with varying levels of comfort and fluency, and this variation can influence how ambitious a module can be at different stages [8-11]. For novice learners, overly complex discussions of identity-sensitive care may become overwhelming if they are introduced before students can manage basic introductions and history-taking structures. For more advanced learners, overly simplified content may feel artificial or insufficiently clinical. Module design should therefore be calibrated carefully so that inclusive communication is taught at an appropriate linguistic level while still remaining authentic to clinical practice [12,13]. This may require scaffolded phrasing, optional enrichment material, or parallel practice tasks that allow learners to engage with the same communication goals at different levels of language complexity.

Standardized patient preparation is another critical component. Because the module depends on realistic and respectful live practice, standardized patients should understand not only the clinical details of the case but also the communication priorities being assessed. Structured standardized patient training is a key ingredient of effective medical Spanish education, as our prior curricular work has shown [12,13]. In this setting, preparation should include attention to names and pronouns, anticipated learner errors, how to respond to assumptive versus nonassumptive questioning, and how to provide useful feedback when appropriate. Cases should also avoid reducing LGBTQ+ identity to a single teaching point. Instead, identity should be integrated into a broader clinical and social context so that the encounter reflects ordinary patient care rather than a narrowly staged diversity exercise [2,12].

Programs should also consider involving Spanish-speaking LGBTQ+ community members directly in the module rather than only teaching about them. Concrete options include recruiting community members to serve as standardized patients, or training existing standardized patients with structured community input; inviting community advisors to review case vignettes, phrase banks, and rubric language for authenticity and regional appropriateness; including guest facilitators or panelists from LGBTQ+ community organizations during live practice or debriefing sessions; and compensating community partners appropriately for their time and expertise. Beyond improving the realism of cases, this participation shifts the curriculum from studying a marginalized community to actively partnering with it, builds durable institutional relationships, and creates a mechanism for ongoing feedback as language and community norms evolve [2,12,13].

Programs should also consider where the module fits within the broader curriculum. An inclusive medical Spanish module can be delivered as a stand-alone workshop, embedded within a systems-based block, or incorporated longitudinally across a larger medical Spanish program [11,12]. The best placement depends on available curricular space and institutional priorities. Embedding the module within existing clinical content may be especially effective because it helps position inclusive communication as part of routine patient care rather than as an isolated topic. This can also make it easier to align language instruction with concurrent clinical skills teaching and reinforce the relevance of the material to patient encounters learners are already studying [11,12].

Finally, implementation should be understood as iterative. Even when a module is grounded in patient-informed findings and supported by prior curricular experience, it will still require refinement based on learner performance, facilitator feedback, and practical constraints [2,12,13]. Challenges may include limited time, uneven attendance, variation in facilitator experience, or learner discomfort with identity-sensitive communication [8-12]. These challenges do not argue against implementation. Rather, they underscore the importance of building in opportunities for revision so that the module remains responsive to both educational realities and patient-informed communication goals. In this context, implementation is most successful when it is treated not as a one-time curricular addition, but as an ongoing process of alignment between patient needs, educational design, and clinical communication training.

## Discussion

### Principal Considerations

This tutorial offers a patient-informed framework for developing an inclusive medical Spanish module to address bias in clinical communication with Spanish-speaking LGBTQ+ patients. Rather than treating this content as a narrow language supplement or a generic diversity add-on, the framework positions it as a structured educational response to a documented communication gap at the intersection of language, identity, culture, and clinical assumptions [2,7,12]. A central implication is that inclusive communication in medical Spanish should be taught as a clinical skill rather than as isolated terminology. Learners need repeated opportunities to apply respectful and nonassumptive language during authentic clinical tasks, including opening an encounter, asking about names and forms of address, taking social and sexual histories, discussing partners and family, and responding to patient cues in real time [2-4,12].

### Comparison With Prior Work

This framework builds on 2 areas of prior scholarship. The first is the medical Spanish education literature, which has shown the value of structured language training, systems-based approaches, and performance-based assessment in preparing learners for Spanish-language patient care [8-13]. The second is the literature on transgender and gender-diverse health education, which has identified substantial gaps in learner preparation and called for more deliberate integration of inclusive content into health professions curricula [14-17]. National survey data further indicate that although most US primary care practices now collect sexual orientation and gender identity information, far fewer provide training in LGBTQ+-affirming care, underscoring the need for educational models that prepare learners before they enter practice [18]. What has been less developed is the integration of these 2 areas in a way that is explicitly informed by the communication priorities identified by Spanish-speaking patients themselves. This tutorial addresses that gap by linking patient-reported findings on exclusion and inclusive language preferences to concrete decisions about module objectives, preparatory work, live practice, feedback, and assessment [2,12]. This approach also aligns with broader calls to strengthen language-concordant care and to recognize communication as a core component of equitable health care delivery [7,10].

### Practical Implications for Educators

For educators, one important implication is that inclusive communication should be embedded throughout the curriculum rather than confined to a brief cultural segment or optional add-on. Integrating this content into preparation, simulation, and assessment helps frame it as part of competent clinical performance rather than as secondary material [2,12]. This tutorial also suggests that technology-enhanced preparation, standardized patient practice, and structured feedback are particularly useful when teaching communication that requires both linguistic precision and relational awareness [8-13]. Beyond assessing individual learners, educators should also plan to evaluate the module itself over successive implementations. Established evaluation frameworks such as the Kirkpatrick model provide a practical structure for this purpose, moving from learner reaction and measured learning to observed behavior change in simulated or clinical settings and, ultimately, patient-level outcomes [19].

### Limitations

This paper is a tutorial, not a report of a new intervention study. It does not present new learner outcomes or multisite implementation data. Instead, it synthesizes prior qualitative findings and prior curricular experience to offer a practical framework for educators [2,12,13]. As a result, institutions adopting this model will still need to adapt it to their own learner population, faculty expertise, curricular structure, and available resources. In addition, because medical Spanish programs vary widely across settings, the specific balance of asynchronous preparation, live teaching, simulation, and assessment may need to be modified in different contexts. Another limitation is that no single module can fully address the broader structural and institutional forces that shape bias in health care and medical education. However, the communication patterns targeted in this framework remain important because they directly affect whether patients feel recognized, respected, and safe during clinical interactions [2].

Two further limitations deserve note. First, the assessment strategies described in this tutorial emphasize immediate formative evaluation; future implementations of this framework would benefit from longitudinal evaluation, such as reassessment of learners’ inclusive communication skills 6 to 12 months after module completion, to measure skill retention and transfer to clinical practice. Second, Spanish-speaking LGBTQ+ patients are not a monolithic population. Patients hold intersecting identities related to race, ethnicity, socioeconomic status, disability, and immigration experience, and for some patients—including members of many Indigenous communities in Mexico and Central America—Spanish is itself a second language, which can add further complexity to the negotiation of gender-neutral terminology. Educators should therefore treat this framework as a starting point to be adapted in partnership with local communities rather than as a uniform template.

### Conclusions

Developing an inclusive medical Spanish module to address bias in clinical communication requires more than adding terminology to an existing lesson. It requires patient-informed curricular design that recognizes how exclusion is often reproduced through routine clinical language and that prepares learners to communicate more respectfully and effectively with Spanish-speaking LGBTQ+ patients. By drawing on prior qualitative findings and prior curricular implementation experience, this tutorial offers a practical framework for educators seeking to integrate inclusive communication, technology-enhanced preparation, simulation-based practice, and performance-oriented assessment into medical Spanish instruction [2,12,13]. A patient-informed approach of this kind may help health professions programs move beyond general statements about inclusion and toward more concrete training in bias-aware clinical communication.

## Supplementary material

10.2196/98398 Multimedia Appendix 1 Sample patient case: inclusive intake.

10.2196/98398 Multimedia Appendix 2 Sample patient case: relationship and sexual history.

10.2196/98398 Multimedia Appendix 3 Facilitator guide and assessment rubric.

## Abbreviations

LGBTQ+: lesbian, gay, bisexual, transgender/transsexual, queer, and other minority sexual orientations and gender identities
