# Supplementary material for: Inclusive Medical Spanish Education to Address Bias in Clinical Communication: Tutorial on Patient-Informed Module Development
Source: JMIR Med Educ. 2026 Aug 21;12:e98398. doi: 10.2196/98398 (PMC13496390; doi:10.2196/98398)
Supplement: Supplementary file 1 [file mededu-v12-e98398-s001.docx]

**Supplementary File 1. Sample Patient Case**

*Forms of Address and Inclusive Intake Language*

# Purpose

This sample case translates the tutorial framework into a brief, adaptable standardized patient or peer role-play activity. It is designed to help learners practice respectful openings, forms of address, and nonassumptive intake language in Spanish without turning the patient identity into the sole focus of the encounter.

| **Suggested learner level** | Novice to intermediate medical Spanish learners; adaptable for advanced learners by increasing spontaneous follow-up questions. |
| --- | --- |
| **Clinical context** | New patient visit in a primary care or community clinic. |
| **Estimated time** | 8-10 minutes for the encounter; 5 minutes for feedback and reflection. |
| **Primary communication focus** | Opening the encounter, asking how the patient wants to be addressed, avoiding automatic gendered forms of address, and using patient-centered intake language. |
| **Secondary focus** | Repairing a linguistic or interpersonal misstep if the learner uses an incorrect form of address or makes an assumption. |

# Learning Objectives

1. Open a Spanish-language clinical encounter using respectful, patient-centered language.
2. Ask how the patient would like to be addressed without relying automatically on *señor*, *señora*, or *señorita*.
3. Use neutral or open-ended language when asking about household, family, and support systems.
4. Demonstrate a basic communication repair phrase if corrected by the patient.

# Patient Profile for Standardized Patient or Peer Actor

| **Name** | Alex Rivera |
| --- | --- |
| **Age** | 32 |
| **Language background** | Spanish-dominant bilingual patient. Comfortable receiving care in Spanish. |
| **Reason for visit** | Establishing care after recently changing insurance and clinics. Reports intermittent fatigue and wants routine preventive care. |
| **Identity-related detail** | Uses the name Alex and prefers not to be addressed as *señor* or *señora*. If asked respectfully, the patient says they prefer to be called Alex. The case does not require a long discussion of gender identity unless the learner makes it relevant or asks appropriately. |
| **Family/support context** | Lives with a partner and one close friend. Has supportive relatives nearby, but does not describe the household using conventional mother/father/spouse categories unless asked in a way that forces those categories. |
| **Affect** | Polite but observant. More open when the learner uses nonassumptive language. Slightly guarded if the learner repeatedly uses gendered assumptions. |

# Learner Task

The learner should begin the encounter, introduce themselves, confirm the patient’s preferred form of address, and complete a brief intake that includes household or support-system questions. The learner does not need to complete a full history and physical examination.

# Information to Reveal During the Encounter

| **If the learner asks...** | **Patient response** |
| --- | --- |
| How the patient would like to be addressed | “Me puede llamar Alex.” |
| Who lives with the patient | “Vivo con mi pareja y una amistad cercana.” |
| Who supports the patient with health decisions | “Mi pareja me ayuda mucho, y también una prima.” |
| Why the patient changed clinics | “Cambié de seguro y quería encontrar una clínica donde me sintiera con confianza.” |
| Whether the patient has concerns today | “Me he sentido con cansancio, pero también quiero hacerme chequeos de rutina.” |

# Expected Communication Behaviors

- Uses the patient’s name rather than defaulting to señor, señora, or señorita.
- Asks how the patient prefers to be addressed before using gendered forms of address.
- Uses open-ended language for household and support systems rather than assuming spouse, mother, father, or conventional family roles.
- Acknowledges correction without defensiveness if the patient redirects the learner.
- Maintains a clinically ordinary tone so the encounter does not become an interrogation about identity.

# Common Learner Errors to Watch For

| **Error pattern** | **Why it matters** | **Facilitator response** |
| --- | --- | --- |
| Repeated use of *señor*/*señora* without checking preference | May misgender the patient or signal that the clinician is relying on visual assumptions. | Ask the learner to restart the opening using the patient’s name or a preference-checking phrase. |
| Assuming “*esposo*/*esposa*” or “*mamá*/*papá*” in intake questions | Narrows the patient’s ability to describe their own relationship and support system. | Prompt the learner to reframe the question using broader terms such as *pareja*, *familia*, *hogar*, or *personas de apoyo*. |
| Turning the encounter into a lesson about identity rather than routine care | May make the patient feel singled out or reduced to identity. | Redirect the learner to the clinical purpose: respectful address, intake, and relevant history. |
| Avoiding the topic completely because of discomfort | Misses an opportunity to establish respectful communication norms. | Ask the learner to practice one short preference-checking question. |

# Feedback Prompts

- Where did the learner use language that allowed the patient to self-describe?
- Where did the learner rely on visual, gendered, or family-structure assumptions?
- Did the learner maintain a normal clinical flow while still being inclusive?
- If a correction occurred, did the learner acknowledge it and continue respectfully?

# Optional Adaptations

- For novice learners, restrict the task to opening the visit and asking about preferred form of address.
- For intermediate learners, add household, family, and support-system questions.
- For advanced learners, add discussion of preventive care, past experiences with care, and communication repair after a misstep.
