# Supplementary material for: Inclusive Medical Spanish Education to Address Bias in Clinical Communication: Tutorial on Patient-Informed Module Development
Source: JMIR Med Educ. 2026 Aug 21;12:e98398. doi: 10.2196/98398 (PMC13496390; doi:10.2196/98398)
Supplement: Supplementary file 2 [file mededu-v12-e98398-s002.docx]

**Supplementary File 2. Sample Patient Case**

*Relationship Language and Sexual History Taking*

# Purpose

This sample case supports practice with relationship language and sexual history taking in Spanish. The case is designed to help learners obtain clinically relevant information without assuming heterosexuality, monogamy, binary gender identity, or conventional reproductive expectations.

| **Suggested learner level** | Intermediate to advanced medical Spanish learners; adaptable for novice learners by limiting the scope to relationship and partner language. |
| --- | --- |
| **Clinical context** | Urgent care, student health, primary care, or community clinic visit. |
| **Estimated time** | 10-12 minutes for the encounter; 5-8 minutes for feedback and reflection. |
| **Primary communication focus** | Nonassumptive partner, relationship, and sexual history questions in Spanish. |
| **Secondary focus** | Anatomy-specific and behavior-specific language when clinically relevant, rather than gender-based assumptions. |

# Learning Objectives

1. Use open-ended Spanish relationship language that allows the patient to describe partners and relationship structure in their own terms.
2. Obtain a focused sexual history using behavior-specific and clinically relevant questions.
3. Avoid assuming partner gender, monogamy, sexual practices, or reproductive goals.
4. Use respectful communication repair if the learner makes an assumption or uses an incorrect term.

# Patient Profile for Standardized Patient or Peer Actor

| **Name** | Marisol Vega |
| --- | --- |
| **Age** | 27 |
| **Language background** | Spanish-dominant patient who prefers Spanish for sensitive health conversations. |
| **Reason for visit** | Requests STI screening after a recent new sexual partner. No severe symptoms. Reports mild concern and wants confidential, respectful care. |
| **Relationship context** | Has one primary partner and recently had sex with another partner. The gender of partners should not be volunteered unless asked in an open-ended and clinically relevant way. |
| **Clinical details** | No fever, pelvic pain, or genital sores. Mild urinary discomfort for two days. Wants testing and counseling. |
| **Affect** | Cooperative but cautious. Becomes more comfortable when the learner asks open-ended questions and explains why sensitive questions are being asked. |

# Learner Task

The learner should explain the need for sensitive questions, obtain a focused sexual history, identify STI testing needs, and counsel the patient at a basic level. The learner should avoid assuming the gender of partners or using relationship terms that force the patient into husband/wife or boyfriend/girlfriend categories.

# Information to Reveal During the Encounter

| **Topic** | **Patient information** |
| --- | --- |
| Reason for testing | The patient had a recent new sexual partner and wants to be checked. |
| Symptoms | Mild urinary discomfort for two days; no fever, pelvic pain, genital sores, or abnormal bleeding. |
| Partners | One primary partner and one recent new partner. If asked openly, the patient says one partner is a woman and one is nonbinary. |
| Protection | Condom use was inconsistent with the recent new partner. |
| Pregnancy/reproductive context | The patient does not want pregnancy-related assumptions. If clinically asked using anatomy- or exposure-specific language, the patient can answer relevant pregnancy risk questions. |
| Confidentiality concern | The patient asks whether the information will remain confidential. |

# Expected Communication Behaviors

- Introduces sensitive questions by explaining their clinical purpose and confidentiality boundaries.
- Uses partner-neutral language such as pareja or parejas before asking more specific clinically relevant follow-up questions.
- Asks about sexual practices, protection, and exposure risk without assuming partner gender.
- Uses anatomy-specific and behavior-specific language when relevant to testing and prevention.
- Avoids equating gender identity with anatomy, sexual behavior, or pregnancy risk.
- Responds respectfully if the patient corrects terminology or clarifies relationship structure.

# Common Learner Errors to Watch For

| **Error pattern** | **Why it matters** | **Facilitator response** |
| --- | --- | --- |
| Assuming the patient has a boyfriend, husband, or male partner | Introduces heteronormative assumptions and may reduce disclosure. | Ask the learner to reframe using partner-neutral relationship language. |
| Asking only “Are you sexually active?” without clinically useful follow-up | May fail to identify relevant exposures, testing needs, or counseling points. | Prompt behavior-specific follow-up questions while maintaining respectful tone. |
| Using gender identity as a shortcut for anatomy or risk | Can be inaccurate and alienating. | Prompt anatomy-specific and exposure-specific language when clinically relevant. |
| Overcorrecting by using vague language that avoids necessary clinical detail | Can undermine clinical accuracy. | Encourage a balance of inclusive phrasing and clear clinical specificity. |
| Failing to repair after an assumption | Leaves the misstep unresolved and may reduce trust. | Ask the learner to acknowledge, correct, and continue without excessive apology. |

# Assessment Notes

This case is best assessed formatively. Faculty or standardized patients should evaluate whether the learner obtained clinically relevant information while maintaining inclusive, nonassumptive phrasing. Linguistic accuracy should be assessed in relation to communicative effectiveness, not as isolated grammar correction only.

# Optional Adaptations

- For novice learners, remove STI counseling and focus only on asking about partners and support systems.
- For intermediate learners, include testing history, protection, and confidentiality.
- For advanced learners, add counseling about testing windows, prevention options, follow-up, and partner notification using inclusive language.
