# Supplementary material for: Inclusive Medical Spanish Education to Address Bias in Clinical Communication: Tutorial on Patient-Informed Module Development
Source: JMIR Med Educ. 2026 Aug 21;12:e98398. doi: 10.2196/98398 (PMC13496390; doi:10.2196/98398)
Supplement: Supplementary file 3 [file mededu-v12-e98398-s003.docx]

**Supplementary File 3. Facilitator Guide and Assessment Rubric**

*Inclusive Medical Spanish Module*

# Purpose

This facilitator guide and rubric are intended to support implementation of an inclusive medical Spanish module focused on bias-aware clinical communication with Spanish-speaking LGBTQ+ patients. The materials may be used with peer role-play, standardized patient encounters, small-group teaching, or formative assessment.

# Facilitator Preparation Checklist

- Review the module objectives and identify which communication behaviors will be emphasized in the session.
- Review core Spanish terms related to forms of address, partner language, family/support systems, sexual history, and communication repair.
- Clarify that the instructional goal is clinically respectful communication, not memorization of a single “correct” inclusive phrase.
- Prepare learners to make mistakes, receive correction, and repair communication without defensiveness.
- If standardized patients are used, orient them to the case goals, expected learner errors, and feedback priorities.

# Core Teaching Principles

| **Principle** | **Application in teaching** |
| --- | --- |
| Patient preference should guide terminology | Teach learners to ask how patients want to be addressed and to use the patient’s own language when clinically appropriate. |
| Neutral language is useful before preferences are known | Encourage terms that do not force gendered or heteronormative assumptions, especially during intake and relationship history. |
| Clinical specificity still matters | Inclusive language should not become vague. Learners should ask clear anatomy-specific, behavior-specific, and risk-specific questions when clinically relevant. |
| Repair is a basic skill | Learners at every level should know how to acknowledge correction and continue respectfully. |
| Feedback should address language and relational effect | Correct grammar and vocabulary, but also discuss whether the phrasing opened or narrowed the patient’s ability to self-describe. |

# Suggested Module Flow

| **Component** | **Approximate time** | **Facilitator focus** |
| --- | --- | --- |
| Prework | 15-30 minutes | Introduce key concepts, high-yield Spanish terms, and examples of inclusive versus assumptive phrasing. |
| Opening mini-lesson | 10 minutes | Frame inclusive Spanish as clinical communication, patient safety, and trust-building. |
| Low-stakes practice | 15-20 minutes | Have learners rehearse short intake, relationship, or repair tasks in pairs. |
| Case-based practice | 10-15 minutes per learner or pair | Use peer role-play or standardized patient cases to apply language in context. |
| Feedback and reflection | 10 minutes | Discuss what language supported patient self-description, what assumptions appeared, and how repair was handled. |

# Phrase Categories for Learner Preparation

| **Category** | **Function** |
| --- | --- |
| Forms of address | Language for asking how the patient prefers to be addressed and avoiding automatic señor/señora assumptions. |
| Relationship language | Language for asking about partners and relationships without assuming gender, marital status, or relationship structure. |
| Family and support systems | Language for asking who is important in the patient’s care without forcing mother/father/spouse categories. |
| Sexual history | Language for asking clinically necessary questions about partners, practices, protection, testing, and risk without heteronormative assumptions. |
| Anatomy-specific language | Language for discussing organs, symptoms, screenings, or reproductive capacity without equating anatomy with gender identity. |
| Communication repair | Language for acknowledging a mistake, accepting correction, and continuing the encounter respectfully. |

# Sample Formative Assessment Rubric

| **Domain** | **Developing** | **Competent** | **Strong** |
| --- | --- | --- | --- |
| Respectful opening and form of address | Uses a greeting but relies on visual assumptions or inconsistent forms of address. | Asks or confirms how the patient wants to be addressed and generally follows the patient’s preference. | Consistently uses patient-centered forms of address and establishes respectful communication from the start. |
| Nonassumptive relationship and family language | Uses gendered or heteronormative terms without confirming relevance. | Uses broader terms such as partner, household, family, or support system when preferences are unknown. | Uses open-ended language naturally and follows the patient’s terminology throughout the encounter. |
| Sexual history and clinical specificity | Avoids sensitive questions or asks them using assumptions about gender, partners, or practices. | Asks relevant questions with mostly inclusive and clinically clear phrasing. | Balances inclusive language with precise, behavior-specific and anatomy-specific clinical questions. |
| Communication repair | Does not recognize or repair a misstep, or becomes visibly defensive. | Acknowledges correction and makes a basic repair. | Repairs clearly, briefly, and respectfully, then continues the encounter without centering the mistake. |
| Spanish language effectiveness | Meaning is frequently unclear or dependent on English. | Spanish is understandable and adequate for the task, with some errors that do not seriously compromise communication. | Spanish is clear, organized, and appropriate to the clinical task and learner level. |
| Patient-centeredness | Completes some tasks but the interaction feels checklist-driven or assumptive. | Maintains a respectful tone and allows the patient to provide relevant information. | Creates an interaction in which the patient can self-describe, ask questions, and participate comfortably. |

# Feedback Guide

- Start with one specific strength related to communication, not only vocabulary.
- Identify one assumption that appeared in the learner’s language, if applicable.
- Ask the learner to revise one phrase so it is more open-ended or clinically precise.
- If a repair occurred, comment on whether it was brief, respectful, and effective.
- End with one concrete next step for the learner’s next encounter.

# Implementation Notes

| **Challenge** | **Practical strategy** |
| --- | --- |
| Mixed learner proficiency | Use the same case with tiered expectations. Novice learners can focus on openings and basic relationship language; advanced learners can add sexual history, counseling, and repair. |
| Limited faculty expertise | Use co-facilitation when possible and provide faculty with case notes, expected learner errors, and rubric language. |
| Limited curricular time | Move terminology review to asynchronous preparation and reserve live time for practice and feedback. |
| No standardized patient resources | Begin with scripted peer role-play or facilitator-led demonstration before moving to standardized patient encounters. |
| Learner discomfort | Frame mistakes as expected in language learning and teach repair as a required clinical communication skill. |

# Recommended Use

The rubric is intended for formative assessment and feedback. Programs may adapt the domains, performance levels, and expectations according to learner proficiency, course structure, and whether the activity is used for practice, standardized patient assessment, or longitudinal curriculum development.
